# Enhancement of Temozolomide Stability and Anticancer Efficacy by Loading in Monopalmitolein-Based Cubic Phase Nanoparticles

**DOI:** 10.1021/acsomega.4c05291

**Published:** 2024-09-02

**Authors:** Ewa Nazaruk, Ewa Gajda, Iza Ziędalska, Marlena Godlewska, Damian Gawel

**Affiliations:** † Faculty of Chemistry, University of Warsaw, Pasteura 1, Warsaw 02-093, Poland; ‡ Department of Cell Biology and Immunology, Centre of Postgraduate Medical Education, Marymoncka 99/103, Warsaw 01-813, Poland

## Abstract

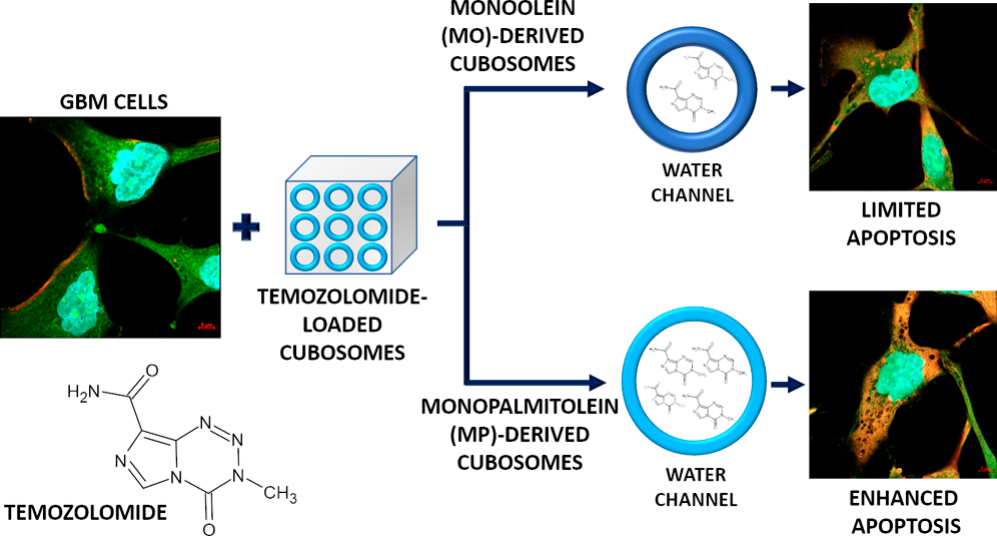

Temozolomide (TMZ) is a prodrug possessing a wide spectrum of anticancer activities. TMZ is pharmacologically inactive, but at a physiological pH, it is quickly converted to an active metabolite, 5-aminoimidazole-4-carboxamide, and a methyldiazonium cation. Due to its chemical nature, TMZ presents some capability of crossing the blood-brain barrier and therefore is used as a first-line agent in the treatment of gliomas. Here, we aimed to improve the anticancer effectiveness of TMZ by loading it into cubosomes, which are lipid nanoparticles recognized as efficient nano-based drug delivery systems. TMZ was incorporated into the monoolein (MO)- and monopalmitolein (MP)-derived cubic phases to improve its stability and half-life. It was considered that the drug release rate may vary between the MO and MP cubosomes, as the water channels of MP phases are larger than those of MO cubosomes. Therefore, we expected that due to the MPs’ ability to entrap more drug molecules inside the mesophase, the concentration of TMZ available to cancer cells would be enhanced. This assumption was supported by biological analyses using the A-172 and drug-resistant T98G glioma-derived cell lines. The strongest reduction in viability was observed for A-172 cells treated with TMZ-loaded MP nanoparticles. Importantly, the TMZ-loaded MPs also caused a significant anticancer effect in the drug-resistant T98G glioma-derived cells. Both MO and MP empty cubic phases did not affect the survival of the tested cells. Concluding, TMZ-loaded cubosomes present strong anticancer properties. Encapsulating the drug within the lipid nanostructure helps to protect the drug from degradation and allows for greater accumulation of TMZ at the tumor site. Together with chemical-based features of mesophases related to increased cargo size and kinetic properties, we imply that MPs may be considered as a highly efficient nano-based drug delivery system to treat poorly curable tumors including gliomas.

## 1 Introduction

The global personalized medicine market in oncology not only requires the development of novel anticancer compounds, such as specific kinase inhibitors presenting antitumor properties, but also advanced molecular-based drug delivery strategies that will increase the effectiveness of transport of encapsulated therapeutic compounds to tumor cells. Currently, the most studied drug delivery systems are non-metal- and metal-based nanoparticles (NPs), as they offer various unique features, including high cargo capacity and the ability to target selected cells, when decorated with proper ligands.^1−3^ One of the most promising and nowadays extensively studied non-metal nanocarriers are cubosomes, which are liquid-crystalline lipidic phases with a cubic inner structure formed by the self-assembly of an amphiphile lipid in excess of water. In the presence of an efficient stabilizer and application of external mechanical energy (high-energy emulsification methods), they form a dispersion. The advantages of these cubic nanostructures arise from their thermodynamic stability, low toxicity, high cargo loading capacity, prolonged drug release, and importantly, their capability to bind both hydrophilic and hydrophobic compounds.^2,4,5^ Cubosomes can be used to enhance the solubility and stability of a wide range of agents, including drugs or peptides.^6,7^ The two main benefits of using cubosomes as delivery vehicles are: (i) their ability to improve the solubility of poor water-soluble medications and (ii) their ability to release the cargo in a controlled and sustained manner. Owing to their superior characteristics, cubosomes can be applied topically, intravenously, orally, intranasally, or ophthalmically. One noteworthy property of cubosomes is their bioadhesiveness, which makes them useful in formulations for topical and mucosal administration of different medications. They also present the potential to bypass the blood-brain barrier (BBB) which is likely related to enhanced drug permeability.^8,9^ Therefore, such nanosized molecular vehicles are of high interest for the treatment of still poorly curable tumors, including glioblastomas (GBMs).^3,10^

**Scheme 1:**
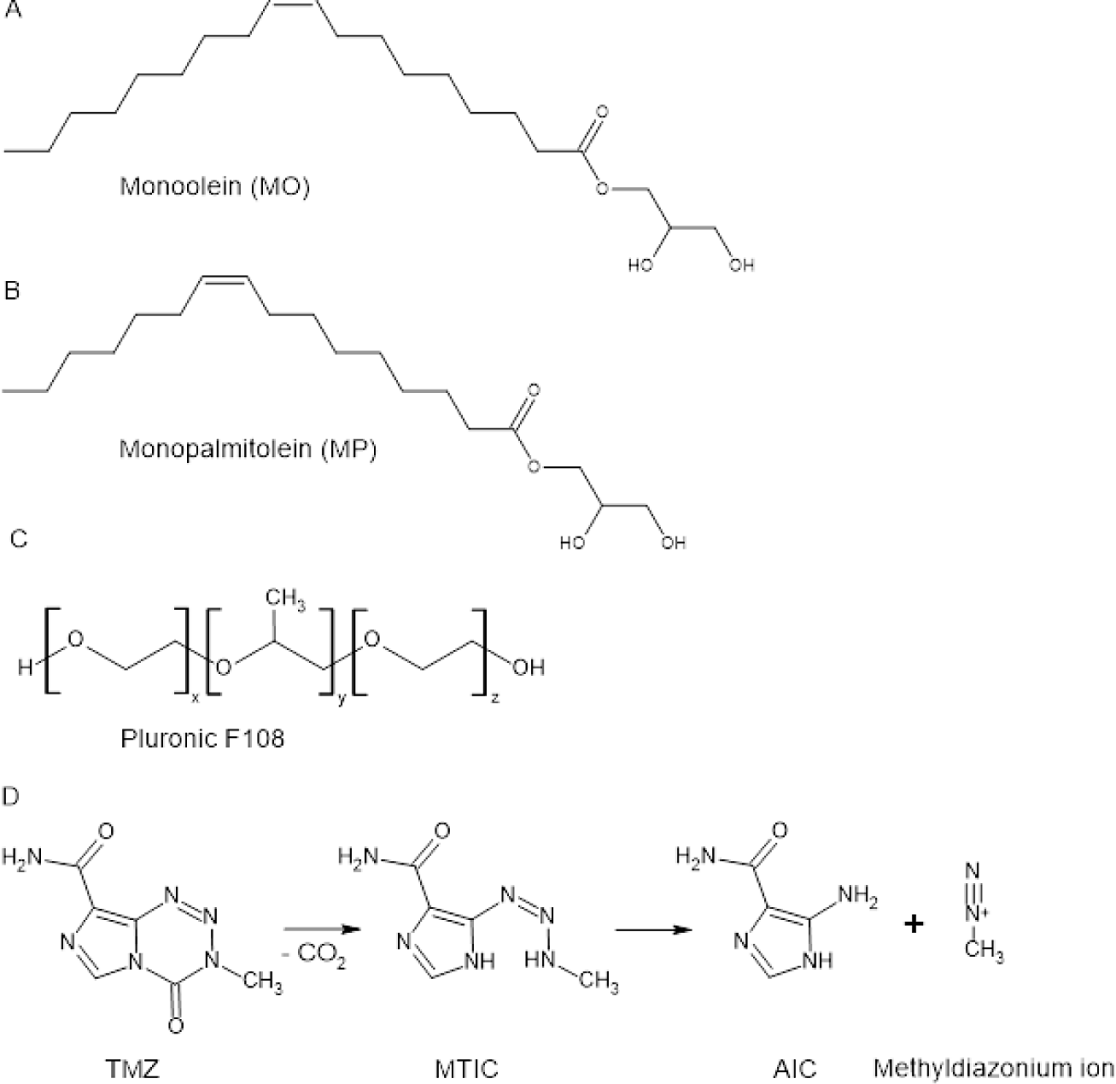
Chemical Structures of the used Reagents A) Monoolein (1-oleoyl-rac-glycerol; MO); B) Monopalmitolein (1-(9Z-hexadecenoyl)-rac-glycerol; C) Pluronic F108; D) Temozolomide (TMZ) and products of TMZ hydrolysis: 5-(3-monomethyl-1-triazeno)imidazole-4-carboxamide (MTIC), 5-aminoimidazole-4-carboxamide (AIC) and a methyldiazonium ion.

As we previously reported, cubic phases formed of monoolein (MO) and loaded with temozolomide, a classic chemo-drug used for the treatment of GBM, present high effectiveness against glioma cells *in vitro*.^10^ Temozolomide (3,4-Dihydro-3-methyl-4-oxoimidazo[5,1-d]-1,2,3,5-tetrazine-8-carboxamide; TMZ) is an alkaline pro-drug that belongs to the imidazotetrazinones and is recommended as a first-line agent for the treatment of high-grade GBM. The structure of TMZ is stable in an acidic pH, but immediately hydrolyses to 3-methyl-(triazen-1-yl)imidazole-4-carboximide (MTIC) in physiological (slightly alkaline; pH of ∼ 7.4) conditions. MTIC is further degraded to 5-aminoimidazole-4-carboxamide (AIC) and a methyldiazonium ion, which is an active methylating agent affecting the O6 position of guanine residues, causing cytotoxic DNA damage (for structures, please see Scheme 1).^11,12^ The small size and lipophilic structure of TMZ enable it to cross the BBB, while in contrast, the transport of the active metabolites of TMZ (MTIC or AIC) through the membrane between the blood and the interstitium of the brain is limited.^13−15^

In our latest research work, we have extended our investigations to encompass an analysis of the stability and cytotoxic properties exhibited by TMZ-loaded cubic phases and cubosomes. We consider that achieving slower TMZ degradation is essential for successful drug delivery to the tumor site, as fast degradation of TMZ in physiological conditions and, in consequence, inability to deliver an effective dose of TMZ to the tumor, limits its therapeutic potential.^16^ To improve the solubility and efficacy of the drug, TMZ was loaded into monoolein and monopalmitolein, two lipid-based NPs that maintain the cubic phase under physiological conditions. MP mesophases are characterized by a larger diameter of aqueous channels in comparison to MO cubosomes. It has been considered that this feature may improve the therapeutic efficacy of MPs, as frequently, the increased loading capability of nanovehicles correlates with enhanced intracellular concentration of the administered drug. We revealed that empty MP phases do not affect the viability of the tested cells, while TMZ-loaded MPs were capable of significantly reducing the survival of both drug-sensitive and drug-resistant glioma-derived cells. Importantly, the observed toxic effect of TMZ-loaded MPs outperformed the effectiveness of both TMZ-loaded MO phases and free TMZ. Therefore, our findings indicate that MP-based vehicles can be considered as preferable carriers for chemotherapeutics in the treatment of cancer when fast drug release and high cargo capabilities are required.

## 2 Materials and Methods

### 2.1 Chemicals and Reagents Used for the Preparation of Cubosomes

Monoolein (1-oleoyl-rac-glycerol; purity ≥ 99%; MO), temozolomide, dimethyl sulfoxide (DMSO), and Pluronic F108 were acquired from Sigma-Aldrich (Sigma-Aldrich, USA). Monopalmitolein (1-(9Z-hexadecenoyl)-rac-glycerol; purity ≥ 99%; MP) was purchased from Jena Bioscience (Jena Bioscience, Germany). All solutions were prepared with Milli-Q water (18.2 MΩ cm^–1^; Millipore, USA).

### 2.2 Preparation of the Monoolein and Monopalmitolein Mesophases

According to the lipid phase diagrams, bulk non-doped cubic phases were formed by combining MP and the acetate buffer solution in a weight ratio of 50:50, and MO and the acetate buffer solution in a weight ratio of 60:40.^17,18^ To form the TMZ-loaded cubic phases, TMZ was dissolved in DMSO and subsequently combined with an acetate buffer solution (pH 5.0) due to TMZ’s low solubility in water. The lipids were melted in a water bath prior to the combination of ingredients. The monoolein was melted at 37 °C, while the monopalmitolein was melted at 40 °C. Following this, the melted lipid was combined with the obtained drug solution. Various concentrations of TMZ in the phases were tested. TMZ-doped MO cubic phases were prepared by mixing 60% by weight of monoolein with acetate buffer solution and varying quantities of the drug (0.1%, 0.2%, and 0.3% by weight of TMZ, respectively). TMZ-doped MP cubic phases were formed by mixing 50% of the weight of the lipid with an acetate buffer solution and 0.3% of the drug. The chemical structures of the compounds utilized in the study are displayed in Scheme 1.

The formulation process for TMZ-loaded cubosomes involved combining 8% cubic phases containing 0.3% TMZ and 92% of a 1% Pluronic F108 solution. To generate cubosomes, the bulk cubic phases underwent fragmentation using SONICS Vibracell VCX 130 (Sonics & Materials Inc., USA) at 40% intensity for 30 min, with 2-s sonic pulses interrupted by 3-s breaks. The same procedure was used to prepare drug-free samples. The final samples maintained a total lipid content of 8%.

### 2.3 Small-Angle X-Ray Scattering

Small-angle X-ray scattering (SAXS) was used to characterize the phase behavior and structural parameters of liquid crystalline phases. Diffraction patterns were recorded using a Bruker Nanostar system working with CuKα radiation, equipped with the Vantec 2000 area detector. For the analysis, the 2D pattern was integrated into the 1D scattering function I(q), where q(Å^-1^) is the length of the scattering vector. The scattering vector q, was determined from the scattering angle using the relationship q = (4π/λ)sinθ, with sinθ being the scattering angle and λ being the wavelength of radiation. Before measurement, the samples were loaded into 1.5 mm capillaries and left to equilibrate at room temperature for at least 12 h. Measurements were performed at 25 °C. The scattered intensity was collected over 5 h for dispersed systems and 10 min for bulk mesophases. The lattice parameters of the mesophases were calculated from the corresponding reciprocal spacings and used to determine structural parameters such as the lipid bilayer thickness and water channel diameter of formulations. Structural parameters of mesophases were determined as described in Supporting Information. Briefly, the average sizes and polydispersity of nanoparticles were determined through the dynamic light scattering (DLS; Zetasizer Nano ZS Malvern, UK) at 25 °C (assuming a viscosity of pure water) and presented as an average of three separate trials. The refractive indexes used for lipid and water were 1.48 and 1.33, respectively. The reported results represent the mean value obtained from three separate trials.

### 2.4 Entrapment Efficiency and Release of Temozolomide

The drug entrapment efficiency of the cubosomes was assessed using the centrifugal ultrafiltration method. Initially, an aliquot of the TMZ-loaded cubosomal dispersion was dissolved in methanol to determine the total concentration of TMZ in the cubosomal dispersion (C_TMZadded_). UV–vis absorbance measurements were utilized to determine the TMZ concentration (Carry 60, Agilent, USA), with methanol serving as the blank solution. The calibration curve was prepared at 329 nm. The amount of drug incorporated into the cubosomes (C_TMZcubosome_) was determined by separating the unbound drug from the cubosomes using Amicon Ultra Centrifugal Filters and a centrifuge (MPW-352R, MPW MED. INSTRUMENTS, Poland). The drug encapsulation efficiency was quantified using the formula: EE% = C_TMZcubosome_/C_TMZadded_ × 100%, where C_TMZcubosome_ represents the concentration of TMZ in the cubosome and C_TMZadded_ represents the concentration of TMZ originally added.

The stability of TMZ in bulk mesophases and its release properties were evaluated using electrochemical methods. Measurements were conducted using a CHI bipotentiostat with a standard three-electrode arrangement in buffered solution. Ag/AgCl was used as the reference electrode and a platinum foil served as the counter electrode. A glassy carbon electrode (GCE) with a surface area of 7.0 mm^2^ that had been modified with TMZ-doped mesophase served as the working electrode. Prior to the experiments, alumina polishing cloths ranging in size from 0.3 to 0.05 μm were used to polish the working electrode. The electrode was then cleaned with ethanol and allowed to air-dry.

Cyclic voltammetry (CV) and differential pulse voltammetry (DPV) were used to study the effect of the tested mesophases on the TMZ-electrode reaction and stability. To determine the release profile from bulk mesophases, the electrode was modified with a TMZ-doped phase. The mesophase was deposited on the electrode surface in the cylindric hole of a Teflon cap, where the electrode surface was exposed to a buffer. The thickness of the mesophase layer was maintained at 0.5 mm so that the geometric volume of this layer remained constant during the experiments. The modified electrode was then immediately immersed in a supporting electrolyte solution, and samples were measured. Before analysis, the samples were deoxygenated by purging with argon (99.999%) for 15 min, and then the argon was passed over the solution surface. A 0.1 M acetate buffer (pH 5) was utilized as the supporting electrolyte due to the fact that at this pH, TMZ does not undergo chemical degradation. The measurements were conducted at room temperature.

The release of TMZ from the monoolein and monopalmitolein cubosomes was determined using a dialysis method. A dialysis membrane with a molecular weight cutoff (MWCO) of 12–14 kDa was employed. Nanoparticles with TMZ were placed in the dialysis membrane, submerged in 50 mL of MES buffer, and magnetically stirred at 50 rpm to obtain the release profile from the dispersed systems. At specific time points, a sample aliquot was withdrawn, and the TMZ concentration was determined spectrophotometrically, as described above. The measurements were conducted at room temperature (ca. 22 °C). The mean value derived from three different trials is represented in the reported results.

### 2.5 Cell Culture

Glioblastoma-derived A-172 and T98G cell lines were purchased from the American Type Culture Collection (ATCC, USA). The T98G cell line was grown in RPMI 1640 medium (HyClone, USA) supplemented with 10% (v/v) fetal bovine serum (FBS; HyClone) and Antibiotic Antimycotic Solution (Sigma-Aldrich). A-172 was maintained in DMEM medium supplemented with 10% FBS (HyClone) and Antibiotic Antimycotic Solution (Sigma-Aldrich) to minimize the risk of microbial growth after the addition of the nanoparticles. All cells were incubated at 37 °C, in a humidified atmosphere containing 5% CO_2_.

### 2.6 Cell Viability (MTS-Based Assay)

The viability of the studied A-172 and T98G cells was measured using the MTS-based assay (CellTiter 96 AQueous One Solution Cell Proliferation MTS Assay; Promega, Germany), as described previously^8^ with some minor modifications. The cells (4 × 10^3^ per well) were seeded in a 96-well plate in 100 μL of complete growth medium and incubated overnight. The next day, the medium was supplemented with: empty nanoparticles (3.2 μL/mL or 6.4 μL/mL); or free TMZ (100 μM or 200 μM); or drug-loaded nanoparticles (3.2 μL/mL or 6.4 μL/mL; TMZ concentration – 100 μM or 200 μM, respectively). Cells not exposed to nanoparticles served as controls. After 24 h, the MTS reagent was added to the wells (20 μL per well) and incubation was continued for an additional 3 h. A microplate reader Synergy2 (BioTek Instruments, USA) was used to measure the absorbance at a wavelength of 490 nm. The results were expressed as a percentage of proliferating cells compared to the controls (100%).

### 2.7 Trypan Blue-Based Dye Exclusion Assay

The viability of the treated cells (A-172 and T98G) was further evaluated using the trypan blue-based assay, as previously described^19^ with minor changes. Briefly, 1 × 10^5^ cells were seeded in each well of a 12-well plate in 1 mL of complete growth medium. After 24 h, the medium was supplemented with: empty nanoparticles (3.2 μL/mL or 6.4 μL/mL); or free TMZ (100 μM or 200 μM); or drug-loaded nanoparticles (3.2 μL/mL or 6.4 μL/mL; TMZ concentration – 100 μM or 200 μM, respectively). Cells not exposed to nanoparticles served as controls. After 24 h of incubation, all the cells were harvested, pelleted by centrifugation (200 × g for 5 min), resuspended in Dulbecco’s phosphate buffered saline (D-PBS; Sigma-Aldrich) and stained with trypan blue (NanoEnTek, South Korea) at the final concentration of 0.2% (w/v) for 10 min. The number of viable and necrotic cells was determined using an EVE Automatic Cell Counter (NanoEnTek) and the results were expressed as a percentage of viable cells compared to the nontreated controls.

### 2.8 Flow Cytometry Analysis of Cell Apoptosis (Annexin V/Propidium Iodide-Based Assay)

The percentages of viable, apoptotic and necrotic cells were determined using flow cytometry. Twenty-four hours prior to the analysis, 1 × 10^5^ of A-172 or T98G cells were seeded in each well of a 12-well plate in 1 mL of complete medium. Next, the growth medium was supplemented with: empty nanoparticles (3.2 μL/mL or 6.4 μL/mL); or free TMZ (100 μM or 200 μM); or drug-loaded nanoparticles (3.2 μL/mL or 6.4 μL/mL; TMZ concentration – 100 μM or 200 μM, respectively). Non-treated cells served as controls. After 24 h, all the cells (adhered and non-attached) were harvested and washed once with D-PBS. Next, the cell pellet was resuspended in 500 μL of Annexin V Binding Buffer (BD Biosciences, USA) and subsequently stained with Annexin V conjugated with fluorescein isothiocyanate (5 μL of Annexin V-FITC; BD Biosciences) and propidium iodide staining solution (5 μL; BD Biosciences). After 15 min of incubation, the analysis was completed using the BD Accuri C6 Plus flow cytometer (BD Biosciences). The data were calculated using dedicated BD Biosciences software (version 1.0.23.1) and expressed as a percentage of viable and apoptotic cells.

### 2.9 Microscopic Analysis of Cell Apoptosis (TUNEL Assay)

To evaluate the levels of apoptosis in the treated A-172 cell lines, the Click-iT TUNEL Alexa Fluor 647 Imaging Assay (Thermo Scientific, USA) was used according to the manufacturer’s protocol with some minor modifications. 1 × 10^5^ of A-172 cells were seeded (per well) on uncoated glass coverslips (ϕ=12 mm, #1.5; ThermoScientific) placed in a 6-well plate in a final volume of 2 mL of complete growth medium. After 24 h of incubation, the medium was supplemented with: empty nanoparticles (3.2 μL/mL); or drug-loaded nanoparticles (3.2 μL/mL; TMZ concentration – 100 μM); or free TMZ (100 μM). Non-treated cells served as a control. After 24 h of incubation, the coverslips were transferred to a clean 24-well plate, washed twice with D-PBS, and fixed with 4% (w/v) paraformaldehyde (ThermoScientific) for 10 min. Cell fixation and further steps were performed at room temperature. After two washes with D-PBS, the cells were permeabilized for 20 min with 0.25% (v/v) Triton X-100 (Sigma-Aldrich) in D-PBS, followed by two washes with deionized water. Then, the cells were incubated with 100 μL of terminal deoxynucleotidyl transferase (TdT) reaction buffer for 10 min, followed by a 1 h incubation with the TdT reaction cocktail (100 μL) in a humidified chamber. After two washes with 3% (w/v) bovine albumin (BSA; Sigma-Aldrich) in D-PBS (2 min each), the coverslips were incubated with the Click-iT reaction cocktail for 30 min in the dark, and washed once with 3% BSA in D-PBS for 5 min. Additionally, the cells were stained with phalloidin-FITC (2 μg/mL in D-PBS; Sigma-Aldrich) for 30 min. For nuclei staining, cells were incubated with 4′,6-diamino-2-phenylindole dihydrochloride (DAPI; 1 μg/mL in deionized water; Sigma-Aldrich), extensively washed with deionized water, and finally mounted in Fluorescence Mounting Medium (Dako, USA). The images were obtained using the Zeiss LSM800 confocal microscope equipped with a plan-apochromatic 63×/1.4 oil DIC M27 objective. The fluorescence from Alexa Fluor 647 and DAPI bound to DNA was measured at λ_ex_650/λ_em_670 nm and λ_ex_340/λ_em_488 nm, respectively. The fluorescence signal from phalloidin-FITC was detected at λ_ex_495/λ_em_520 nm. The images were analyzed with dedicated ZEN 2.1 software (Zeiss, Germany) and saved in TIFF format.

### 2.10 Biological Data Analysis

Graphical and statistical analysis of the data was completed using Prism software (version 6.0; GraphPad, Inc., USA). One-way ANOVA and post-hoc Bonferroni’s multiple comparison tests were used for statistical analysis. All the experiments were performed at least three times. Statistical significance was considered at *p* < 0.05. Data on figures were presented as mean ± SD (standard deviation).

## 3 Results and Discussion

### 3.1 Phase Behavior and Characterization of TMZ-Loaded Mesophases

TMZ-loaded mesophases were characterized using SAXS. Monopalmitolein and monoolein were selected to form TMZ-loaded cubic phases under full hydration conditions. Monopalmitolein, a monoacylglycerol with a shorter hydrophobic tail than monoolein, when fully hydrated, forms cubic phases that present a larger aqueous channel diameter. The increased size of the water channel may affect the transport of guest molecules, as the larger channels might facilitate easier inclusion and faster release of drug molecules.

**Figure 1:**
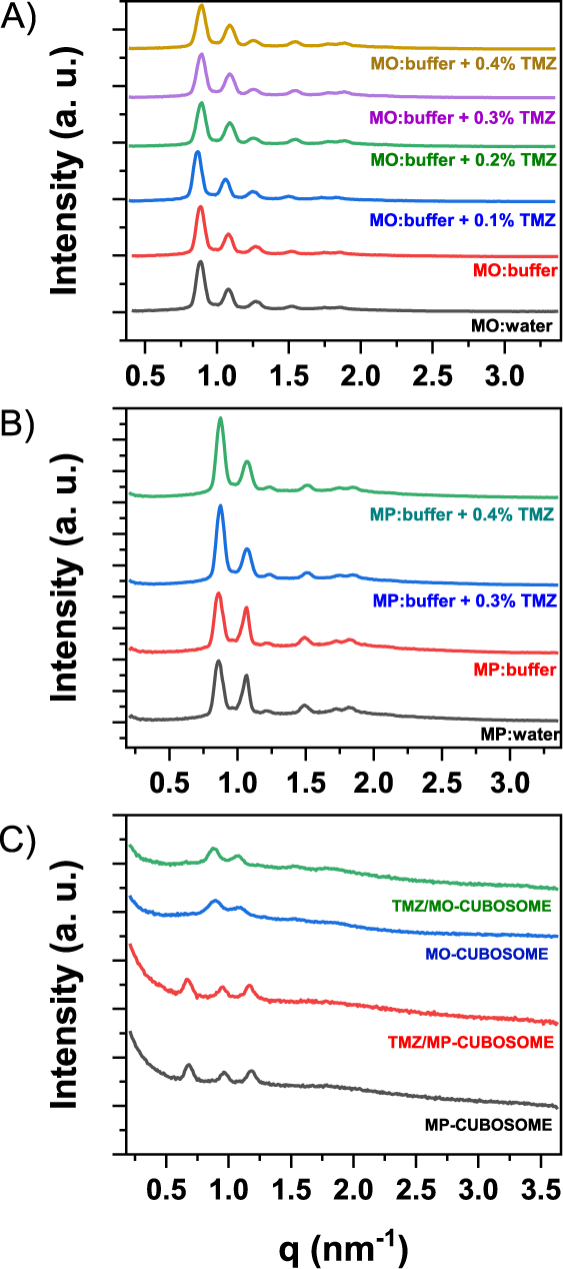
A) Diffractograms of cubic phases prepared from monoolein (MO) and acetate buffer, doped with various concentrations of temozolomide (TMZ); B) diffractograms for cubic phases prepared from monopalmitolein (MP) and temozolomide; C) diffractograms of cubosomes prepared from MO or MP and acetate buffer, doped with TMZ.

Four concentrations of TMZ in the range from 0.1 wt % to 0.4 wt % were prepared to investigate the effect of the drug concentration on the symmetry and structural parameters of the mesophases. The drug was observed to precipitate in the 0.4% TMZ phase, so this phase was not used in the following steps to determine the release profile. Nevertheless, the structure was inspected to confirm that the compound in higher concentrations did not alter the symmetry. The 1D scattering patterns obtained from MO and MP doped with increasing amounts of TMZ hydrated in buffer, and diffractograms of the fully hydrated bulk phase are shown in Figure 1. The second lipid, MP, characterized by wider water channels, was doped with 0.3% by weight of TMZ to compare it to the monoolein phase containing 0.3% by weight of the drug.

The SAXS measurements were conducted to investigate whether incorporation of TMZ alters the internal structure of the tested mesophases. Six signals were observed in each diffraction pattern. The assignment of the appropriate Miller indexes to the signals enabled determination of the structure of the obtained phases. The positions of the diffraction peaks in the integrated curve correspond to the crystal plane reflections with Miller indices (hkl) = (110), (111), (200), (211), (220), (221). Figure 1 shows the set of Bragg peaks with q-vector positions spaced in the ratio of √2:√3:√4:√6:√8:√9, indicating a cubic lattice. Figure S2 illustrates the indexing of the X-ray diffraction (SAXS) data for the MO and MP cubic phases.

For cubic phases, the lattice constant (a) was calculated using the formula: . The MO formed cubic Pn3m phases with a lattice parameter of *ca.* 10 nm. The lattice parameter allowed to determine the structural properties of the cubic phases, such as the diameter of aqueous channels and lipid length. Table 1 presents the assigned mesophases and the structural parameters calculated from the collected SAXS diffraction patterns. For all samples, cubic phases of Pn3m symmetry were obtained, with a larger aqueous channel width for the MP-derived formulations.

**Table 1:** Parameters for Temozolomide-Loaded Monoolein- and Monopalmitolein-derived Pn3m Cubic Phases

	Lattice parameter a [nm]	Water weight fraction φ_w_	Lipid weight fraction φ_l_	Lipid length l [nm]	Water channel diameter d [nm]
MO:water	9.8	0.39	0.61	1.7	4.3
MO:buffer pH 5.0	9.8	0.39	0.61	1.7	4.3
MO:buffer pH 5.0 + 0.1% TMZ	10.0	0.39	0.61	1.7	4.4
MO:buffer pH 5.0 + 0.2% TMZ	10.0	0.39	0.61	1.7	4.4
MO:buffer pH 5.0 + 0.3% TMZ	10.0	0.39	0.61	1.7	4.4
MO:buffer pH 5.0 + 0.4% TMZ	10.1	0.39	0.61	1.7	4.5
MP:buffer pH 5.0	10.2	0.51	0.49	1.4	5.2
MP:buffer pH 5.0 + 0.3% TMZ	10.3	0.51	0.49	1.4	5.3

Cubic phases exist in equilibrium with excess water and can be dispersed to form cubosomes. The ability of cubic phases to exist as dispersed particles is the most meaningful. In the diffraction patterns of cubosomes with MO, six signals were observed, and the ratios of successive peaks were √2:√3:√4:√6:√8:√9, which is characteristic of Pn3m symmetry. In the case of MP cubosomes, three signals were observed, and the ratios of successive peaks were √2:√4:√6, which indicates Im3m symmetry. Identically as in the case of phases, the crystal lattice parameter, lipid length, radius, and diameter of aqueous channels in cubosomes were determined (Figure 1C). After fragmentation, the MO cubosomes retained the parent cubic Pn3m symmetry, while the MP cubosomes underwent transition to the cubic Im3m symmetry. This transition may be the result of interactions of the lipid with Pluronic F108, which was used to stabilize the nanoparticles. The addition of TMZ slightly widened the water channels in MO and MP cubic phases. Based on the data in the table above, it can also be concluded that the water channels of MP cubosomes are approximately 1 nm larger in diameter than those of MO cubosomes. The calculated values are presented in Table 2.

**Table 2:** Phase and Lattice Parameters Measured using Small-angle X-ray Scattering. Size Distribution, Zeta Potential and Polydispersity Index were Obtained using Dynamic Light Scattering

Formulation	Symmetry	Lattice parameter a [nm]	Water channel diameter d [nm]	Size [nm]	Polydispersity index (PDI)	Zeta potential [mV]	Entrapment efficiency EE [%]
MO (empty)	Pn3m	10.0	4.3	162 ± 23	0.14	-29 ± 0.9	----------
MO/TMZ	Pn3m	10.2	4.6	174 ± 25	0.15	-30 ± 1	97%
MP (empty)	Im3m	13.1	5.2	178 ± 43	0.17	-27 ± 0.8	----------
MP/TMZ	Im3m	13.5	5.5	189 ± 28	0.23	-29 ± 0.5	98%

Physicochemical characterization of cubosomes was achieved using DLS. The size distribution and polydispersity index (PDI) values of the particles are summarized in Table 2. The formulations presented size distribution < 200 nm. All the samples displayed PDI values < 0.2. The zeta potentials for all formulations were close to −30 mV. In addition, the effectiveness of TMZ immobilization was determined using a spectrophotometric method and shown as entrapment efficiency.

### 3.2 Electrochemical Characterization of TMZ Incorporated into Lipid Mesophases—In Vitro Release Study

As TMZ presents a short half-life in blood plasma (< 2 h), it requires to be stabilized.^20^ Entrapment of the drug into the lipid cubic mesophase may improve drug stability and extend the half-life of TMZ *in vivo* by protecting the drug from degradation. Additionally, a greater interfacial surface area of the cubic phase (*ca*. 400 m^2^/g) permits for high cargo capacity, which allows for an increase in the concentration of TMZ available to cancer cells.^21^

**Figure 2:**
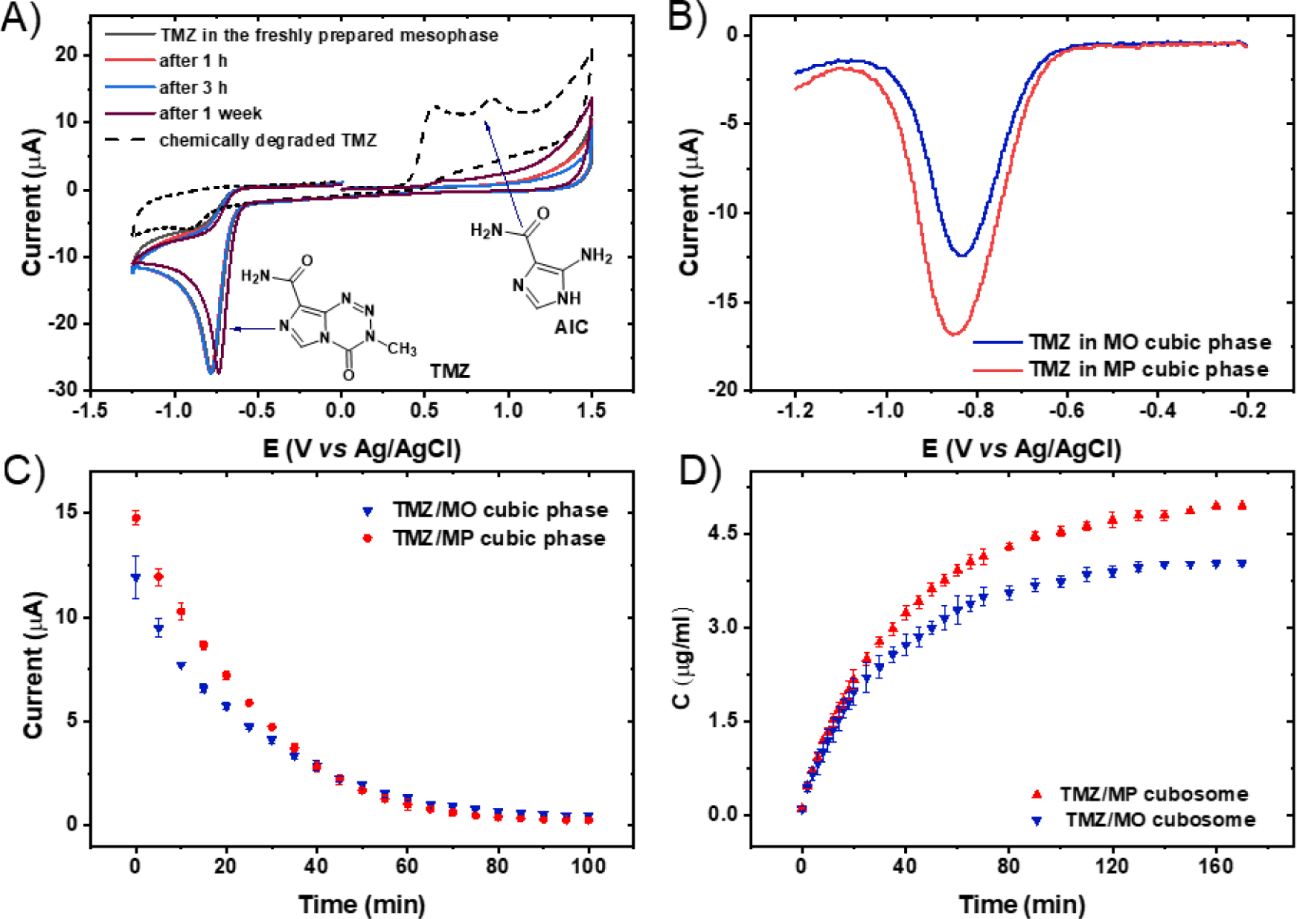
A) Cyclic voltammogram recorded on the glassy carbon electrode (GCE) for TMZ in an acetic buffer (pH of 5.0); scan rate 50 mV s^–1^; B) Differential pulse voltammograms recorded on the GCE for the MO and MP cubic phases; amplitude: ΔE = 50 mV, pulse time: tp = 50 ms; C) Drug release profile for TMZ-loaded MO and MP cubic phases; D) TMZ release profile from cubic phase nanoparticles.

Here, electrochemistry was applied to define prodrug stability in cubic phases and evaluate the metabolites of TMZ in an aqueous solution. The redox properties of temozolomide have already been described by Lopes et al. and it was shown that the decomposition of TMZ can be monitored using electrochemical methods.^22,23^ Chemical degradation of TMZ can be evidenced electrochemically by the appearance of: (i) an irreversible anodic peak at + 0.5 V, which corresponds to the irreversible oxidation of the tetrazin ring, and (ii) a peak at + 0.8 V that can be attributed to the irreversible oxidation of the nitrogen in the already-open tetrazin ring.^23^ Cyclic voltammogram recorded on a GCE in deoxygenated acetic buffer solution (pH 5.0) for TMZ incorporated into the monoolein cubic phase was shown in Figure 2A. On the voltammogram a reduction peak was observed at −0.8 V, which is related to the reduction of the tetrazin ring. No significant changes in the voltammogram were found for the TMZ-loaded mesophase after 1 week of storage, meaning that TMZ does not undergo degradation to its derivatives (MTIC or AIC) within this time frame. This behavior was observed for both MO and MP cubic phases. Due to better stability and reproducibility, the reduction peak at −0.8 V was selected to determine the drug release profile. Additionally, the stability of the reduction peak (at −0.8 V) with respect to various pH values is shown in Figure S1.

The TMZ release profile for the monoolein and monopalmitolein bulk cubic phases was determined using DPV. The microenvironment of proliferative and aggressive tumors, such as the GBM, is often acidic with a pH of 6.8 or lower.^24^ Thus, for the study, only pH 5.0 was selected for release. The recorded DPVs (shown in Figure 2B) present the plots obtained for both mesophases loaded with 0.3% by weight of TMZ. The current potential graph shows that the initial current for the MP phase was higher than the initial current for the MO phase, which may be related to the structural parameters of the mesophases. As the larger aqueous channel size of MPs is favorable for the entrapment of more drug molecules inside the mesophase, the MP-based cubic phase is beneficial for TMZ accumulation. Moreover, the size of the aqueous channels may affect the diffusion and transport of drugs. It was observed that the release profile from mesophases exhibited comparable release properties, nevertheless, a slightly faster release was observed for the MP-derived formulation (Figure 2C).

Next, the release of TMZ from the MO- and MP-based cubosomes was determined. We employed differential pulse voltammetry for this purpose, following the procedure outlined in our earlier research.^8,25^

The release profiles of TMZ-loaded cubosomes followed a similar pattern as those from the bulk cubic phase (Figure 2D). The release was saturated after about 70 min, and the drug release from the MP cubosomes was higher than from the MO cubosomes. The TMZ release profiles from the MO- and MP-derived cubosomes exhibited a relatively fast release rate. As for the bulk system, the TMZ release rate was affected by the size of the channels and the drug release was found to be higher in the MP-based cubosomal dispersion in comparison to the MO cubosomes. MP-based cubosomes displayed Im3m symmetry, where slower transport was expected compared to the more porous Pn3m geometry. However, it was shown that cargo diffusion is determined mainly by the size of aqueous channels and that the geometry does not impact diffusion.^6^

To determine the effect of the lipids on the kinetics of release, TMZ release data from the MO and MP cubic phases and cubosomes were fitted into diffusion models and analyzed. The results from these analyses are shown in Table 3. Figure S3 illustrates the percentage of accumulated drug released versus time to determine the release kinetics. The value of the exponent “n” in the Korsmeyer-Peppas model was calculated as an indicator of the drug transport mechanism (as described in the Supporting Information; S2). MO-derived cubic phases with incorporated TMZ showed “n” values around 0.6, indicating anomalous drug transport. The drug release mechanism was a result of the contribution of the Fickian diffusion and relaxation. The diffusional exponent was slightly larger for the MP-based cubic phase. This could probably be due to additional interactions of the drug with MO, the host lipid. A similar effect was observed for the dispersed systems. The Peppas-Sahlin model was then used to identify the contribution of diffusional and relaxational mechanisms in drug release.

**Table 3:** Summary of Elution Profiles from the Monoolein and Monopalmitolein Phases Containing the Same Concentration of Temozolomide Fitted to the Kinetic Models

	I order model	Higuchi model	Korsmeyer-Peppas model		Peppas-Sahlin model			
	R^2^	R^2^	n	R^2^	k_1_	k_2_	m	R^2^
TMZ/MO cubic phase	0.996	0.996	0.60	0.995	53.9	45.0	0.41	0.994
TMZ/MP cubic phase	0.995	0.993	0.71	0.999	30.4	88.5	0.46	0.999
TMZ/MO cubosome	0.995	0.994	0.65	0.993	40.8	64.8	0.50	0.999
TMZ/MP cubosome	0.999	0.998	0.70	0.997	7.5	88.1	0.37	0.999

**Figure 3:**
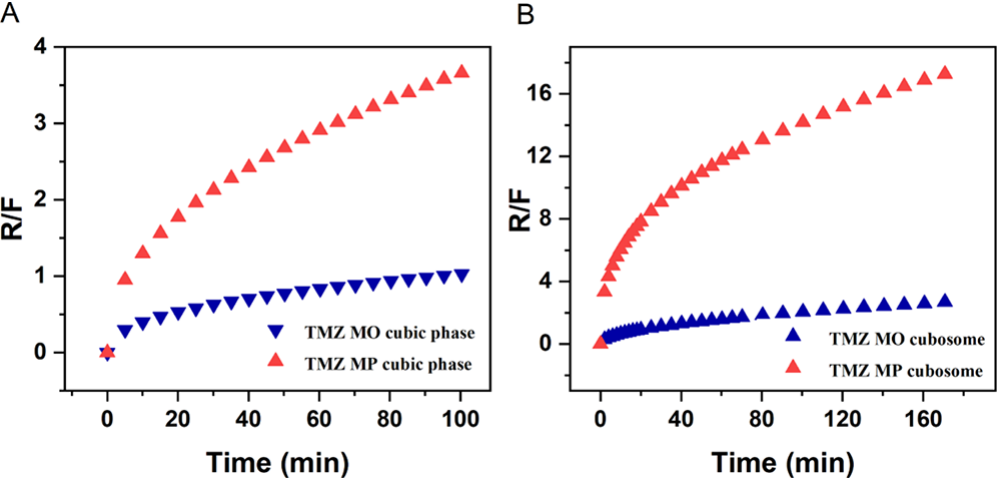
Ratio of the relaxation input (R) and the diffusion input according to Fick (F) (R/F ratio) for TMZ release from the MO- and MP-based A) cubic phase and B) cubosomes.

The amount of drug released by the diffusion mechanism was calculated as . The ratio of the drug released by relaxation to the amount of drug released by diffusion showed the relationship . The ratio of the relaxation input (R) and the diffusion input according to Fick (F) was calculated for all systems (Figure 3). R/*F* > 1 indicated that the relaxational contribution was more predominant than the diffusional contribution. The initial drug release from the mesophases was controlled by the Fickian diffusion. The increase in the R/F ratio with time indicated the increasing relaxational contribution and for MP-based cubosomes, the relaxation contribution was considerable.

### 3.3 Analysis of the Properties of TMZ-Loaded Mesophases

**Figure 4:**
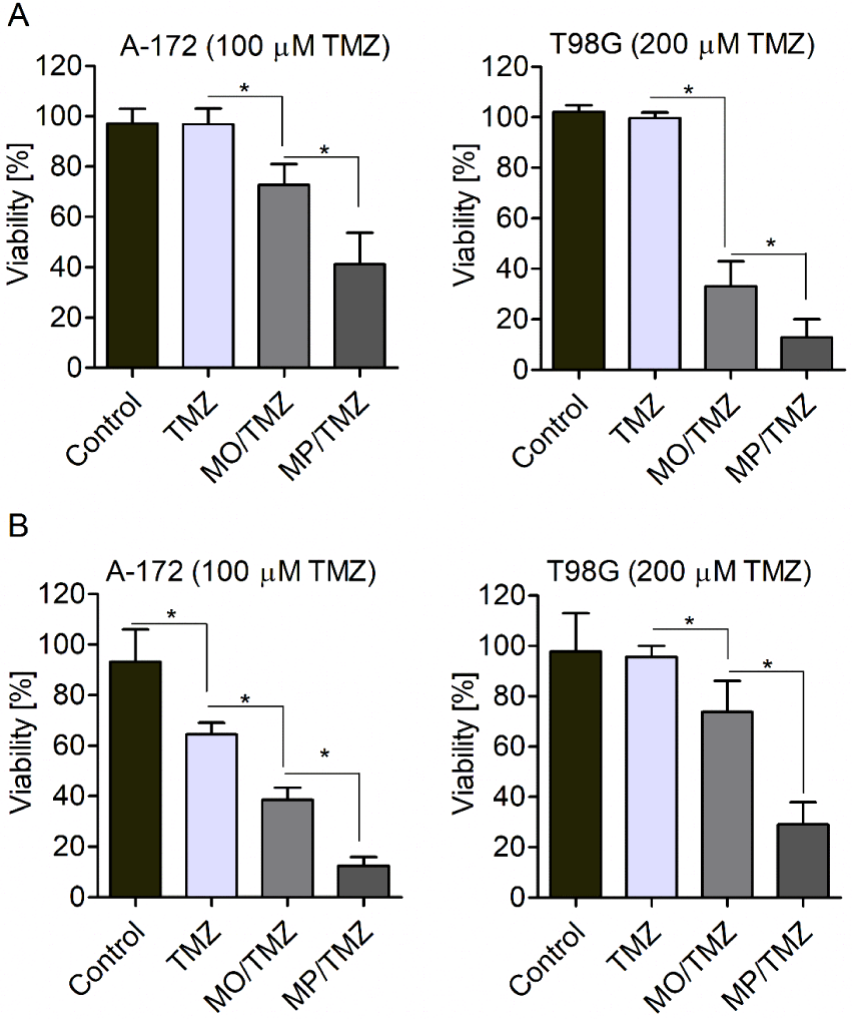
Analysis of the survival rate of A-172 and T98G cells treated with free TMZ (TMZ) and TMZ-loaded MO or MP phases (MO/TMZ; MP/TMZ, respectively). The cell’s viability was measured using MTS (A) and trypan blue exclusion (B) assays. Data are presented as mean ± SD (standard deviation); (*n* = 8). Non-treated cells served as controls. * *p* < 0.05.

**Figure 5:**
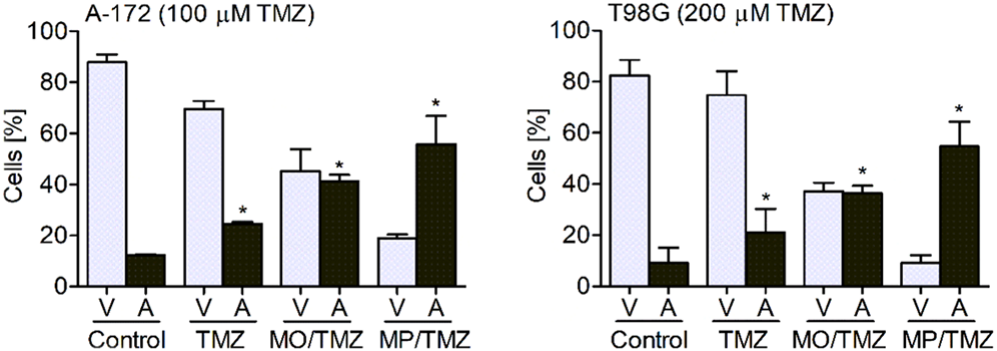
Flow cytometry analysis of A-172 and T98G cells treated with free TMZ and TMZ-loaded MO or MP phases. The light color bars show the number of viable cells (V), while the dark color bars refer to the number of apoptotic cells (A) in each of the tested samples. Data are presented as mean ± SD; (*n* = 10). Control—non-treated cells. * *p* < 0.05.

To evaluate the properties of the tested phases, a side-by-side comparison analysis of TMZ-loaded MO and MP particles was performed. We were especially interested in evaluating the toxicity of empty formulations, and most importantly, assessing the anticancer potential of drug-loaded MPs, as the chemical analysis indicated their greater relaxation and larger aqueous channel diameter. In the experiments, drug-sensitive A-172 and drug-resistant T98G glioma-derived cell lines were used. The analysis of the tested phases was performed using two classical assays: MTS and trypan blue staining. Initially, both cell lines were exposed to various doses of non-loaded nanoparticles to establish an optimum working concentration of non-loaded carriers, which would not inhibit the growth of the tested cells (Figure 4). It was also determined that the 100 μM concentration of free TMZ does not significantly affect the survival rate of the drug-sensitive A-172 cells (Figure 5). In the case of the drug-resistant T98G cell line, the 200 μM TMZ concentration was found to be non-harmful to the cells (Figure 4). Importantly, it was established that the A-172 and T98G cells treated with 3.2 μL/mL of non-loaded MO or 6.4 μL/mL of non-loaded MP phases present similar, high viability in all of the applied assays (Figure S4). This confirms our previous observations^23^ indicating that the toxicity of empty cubosomes in the tested concentrations is limited. Therefore, both MP and MO formulations can be considered as non-harmful for cells.

The analyses of the effect of TMZ-loaded nanoparticles on the survival of the tested cells revealed that cells exposed to free TMZ did not exhibit a significantly altered survival rate. In contrast, it was found that both of the tested cell lines exposed to TMZ-loaded MO or TMZ-loaded MP formulations (MO/TMZ; MP/TMZ, respectively) presented significantly decreased viability. This phenomenon was observed in both the MTS and trypan blue exclusion assays (Figure 4A,4B, respectively).

These results indicate that exposure of cells to the same concentration of the drug can be more effective when TMZ is entrapped in MO or MP channels and further delivered to the cells. Exposing the cells to free TMZ showed no effect on tumor cells. Likely, the lack of an anticancer effect of free-administered TMZ arises from limitations of the diffusion process, which include a very slow approach to apparent equilibrium. Additionally, such prolongated drug delivery allows the targeted cells to induce various stress response mechanisms and factors, including multidrug (MDR) efflux pumps (as shown previously^10^), which can actively prevent cells from accumulating a toxic concentration of the therapeutic agent. Therefore, it can be considered that increased drug delivery within cubosome phases may also limit cancer cell response systems relevant for drug intake (as already presented for MO.^26^ Importantly, it was found that the viability of the analyzed glioma cells was most significantly altered when TMZ-loaded MP phases were applied. It was considered that the strong antitumor effect of the MP/TMZ mesophases might be a consequence of the chemical properties of the MP phase, which warrants higher cargo capacity than MO and faster release of the drug from nanochannels due to altered kinetic properties.

The observed chemical and biological properties of the MP/TMZ mesophases were further supported by cytometric analysis. It was revealed that even though the number of viable cells was significantly reduced in cell lines treated with both drug-loaded lipids. Importantly, TMZ-loaded MPs presented a more toxic effect (measured as a percentage of apoptotic cells) (Figure 5).

Interestingly, it was observed that the number of apoptotic cells only modestly varied between the MO/TMZ- and MP/TMZ-treated cells, while the number of viable cells was decreased. This indicates that in the samples treated with MP/TMZ, nearly 50% of the cellular population was necrotic, while in the samples treated with MO/TMZ, only 20% of cells were dead. This data supports a model in which the drug-loaded MP mesophases are capable of fast release of cargo, which results in rapid cell damage, followed by cell death.

**Figure 6:**
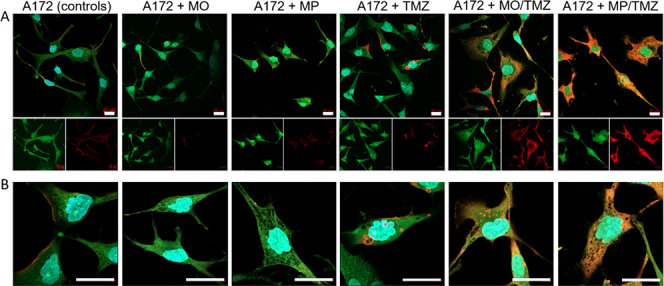
Click-iT TUNEL Alexa Fluor 647 Imaging Assay of A-172 cells exposed to free TMZ, TMZ-loaded MO or TMZ-loaded MP phases for 24 h (representative images). Non-treated cells or cells treated with non-loaded MO or non-loaded MP particles served as controls. The intensity of the red signal (Alexa Fluor 647) refers to the apoptotic rate in the tested cells. The green signal (phalloidin-FITC) indicates the organization of the cell structure. Cell nuclei were counterstained with DAPI (blue signal). Objective: 63x/1.4 oil DIC M27. Panel A – standard confocal imaging; Panel B – Airyscan imaging. Scale bar = 20 μm.

Finally, the toxicity of the MO/TMZ and MP/TMZ nanoparticles was confirmed using confocal imaging. The TUNEL assay revealed that cells treated with nonloaded MO- and MP-phases were not affected, in comparison to the control cells. Additionally, exposure of cells to free TMZ also did not result in significant changes in cell organization or apoptosis. This can likely be linked with the poor tumor cell infiltration capabilities of free TMZ. Similarly to the data from flow cytometry, the apoptotic process in MP/TMZ-treated cells was found to be the most advanced, when compared to cells treated with MO/TMZ (Figure 6). This observation also supports the model in which increased drug release/cell penetration is directly linked with the chemo-physical properties offered by the MP formulation.

## 4 Conclusion

Temozolomide is a prodrug that possesses strong anticancer activities, although limitations in its antitumor efficacy arise from its reduced stability in physiological conditions. In this study, TMZ was incorporated into both MO- and MP-derived cubic phases. Determination of the profile and kinetics of drug release was carried out at a pH of 5.0 and the release of TMZ was found to be faster in the MP-based cubic phase, in comparison to the MO system. We concluded that likely the observed advantages in the release profile of MP might be related to the observed differences in the structural parameters of both systems, where the aqueous channel diameter is larger for the MP-derived formulation. Moreover, the biological studies revealed that the TMZ-loaded MP formulation presents stronger anticancer properties than the drug-loaded MO phase. What is crucial, the improved effectiveness of MP/TMZ was also observed during treatment of drug-resistant glioma-derived cells. The obtained data indicate that TMZ encapsulated in MPs demonstrates promising therapeutic features and may be considered as an efficient nanodrug shuttle system in personalized medicine. As MP-based formulations present increased TMZ stability and high drug effectiveness, the MP/TMZ cubosomes may offer a new strategy to overcome the restrictions related to the BBB. Nevertheless, more studies are needed to further elucidate the features of cubosome-based drug delivery to the brain.

## Supporting Information Available

Equations used to calculate the mesophase parameters; **Figure S1.** Stability of TMZ reduction current in time; **Figure S2.** Indexing of the X-ray diffraction (SAXS) data of the MO and MP cubic phases; **Figure S3.** Plot of % cumulative drug release vs time; **S2.** Kinetic models; **Figure S4.** Analysis of the survival rate of A-172 (drug-sensitive) and T98G (drug-resistant) cells treated with nonloaded MO or MP phases determined using (A) MTS and (B) trypan blue exclusion assays. The number of viable, nontreated, cells was used as a control (100%). Data are presented as mean ± SD (standard deviation); (*n* = 8). For all analyzed comparisons *p* > 0.05 (PDF)

## Abbreviations

AIC: 5-aminoimidazole-4-carboxamide
ATCC: American Type Culture Collection
BBB: blood-brain barrier
CV: cyclic voltammetry
DAPI: 4’,6-diamino-2phenylindole dihydrochloride
DLS: dynamic light scattering
D-PBS: Dulbecco’s phosphate buffered saline
DPV: differential pulse voltammetry
EE%: encapsulation efficiency
FITC: fluorescein isothiocyanate
GBM: glioblastoma
GC: glassy carbon
GCE: glassy carbon electrode
MO: monoolein
MP: monopalmitolein
MTIC: 3-methyl-(triazene-1-yl)imidazole-4–4-carboximide
PDI: polydispersity index
SAXS: small-angle X-ray scattering
SD: standard deviation
TMZ: temozolomide
