# Supplementary material for: Enhancement of Temozolomide Stability and Anticancer Efficacy by Loading in Monopalmitolein-Based Cubic Phase Nanoparticles
Source: ACS Omega. 2024 Sep 2;9(37):38936–45. doi: 10.1021/acsomega.4c05291 (PMC11411539; doi:10.1021/acsomega.4c05291)
Supplement: Supplementary file 1 — ao4c05291_si_001.pdf [file ao4c05291_si_001.pdf]

Supporting Information

Enhancement of temozolomide stability and anti-cancer efficacy by loading in monopalmitolein-based cubic phase nanoparticles

Ewa Nazaruk[1], Ewa Gajda[2], Iza Zięsdalska[1], Marlena Godlewska[2], Damian Gaweł[2]**

[1] University of Warsaw, Faculty of Chemistry, Pasteura 1, 02-093 Warsaw, Poland

[2] Centre of Postgraduate Medical Education, Department of Cell Biology and Immunology, Marymoncka 99/103, 01-813 Warsaw, Poland

* Authors to whom correspondence should be addressed:

enaz@chem.uw.edu.pl (EN); damian.gawel@cmkp.edu.pl (DG)

KEYWORDS

monopalmitolein; monoolein; cubosomes; temozolomide; glioma cells

S1. Equations used to calculate the mesophase parameters

In order to characterize the structural parameters, the following calculations were performed:

a) lattice parameter (a)

$$a = \frac{2\pi\sqrt{h^2 + k^2 + l^2}}{q_0}$$

where: q0 – value for the first peak in the diffraction pattern; hkl – Miller indices for the first peak in the diffraction pattern;

b) weight fraction of water (φ_w)

$$\varphi_w = \frac{m_w}{m_w + (1 - m_w)\frac{d_w}{d_l}}$$

where: mw – weight of the water fraction, dw – density of the water phase (0.997 g/cm3), dl – lipid density (0.942 g/cm3 for monoolein or 0.982 g/cm3 for monopalmitolein)

c) weight fraction of the lipid (φ_l)

$$\varphi_l = 1 - \varphi_w$$

d) lipid length (l)

$$\varphi_l = 2\sigma\left(\frac{l}{a}\right) + \frac{4}{3}\pi\chi\left(\frac{l}{a}\right)^3$$

where: σ – constant value of 1.919 for the Pn3m phase; χ – constant value of -2 for the Pn3m phase

e) the radius of the water channel (r_w)

$$r_w = \left(-\frac{\sigma}{2\pi\chi}\right)^{1/2} a - l$$

Figure S1. Stability of TMZ reduction current in time.

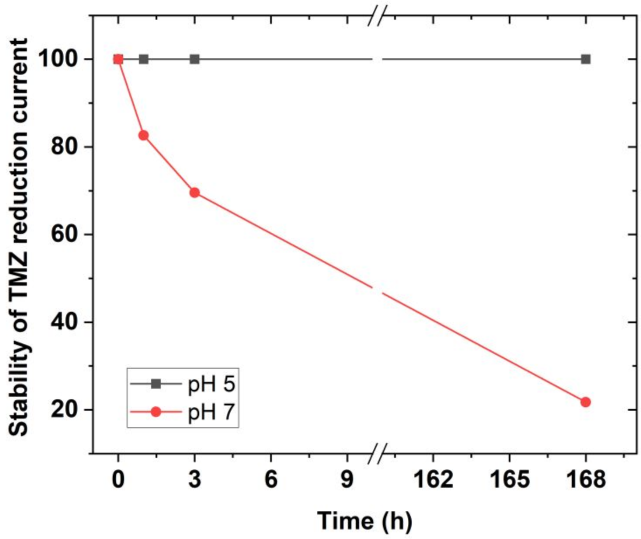

Figure S2. Indexing of the X-ray diffraction (SAXS) data of the MO and MP cubic phases.

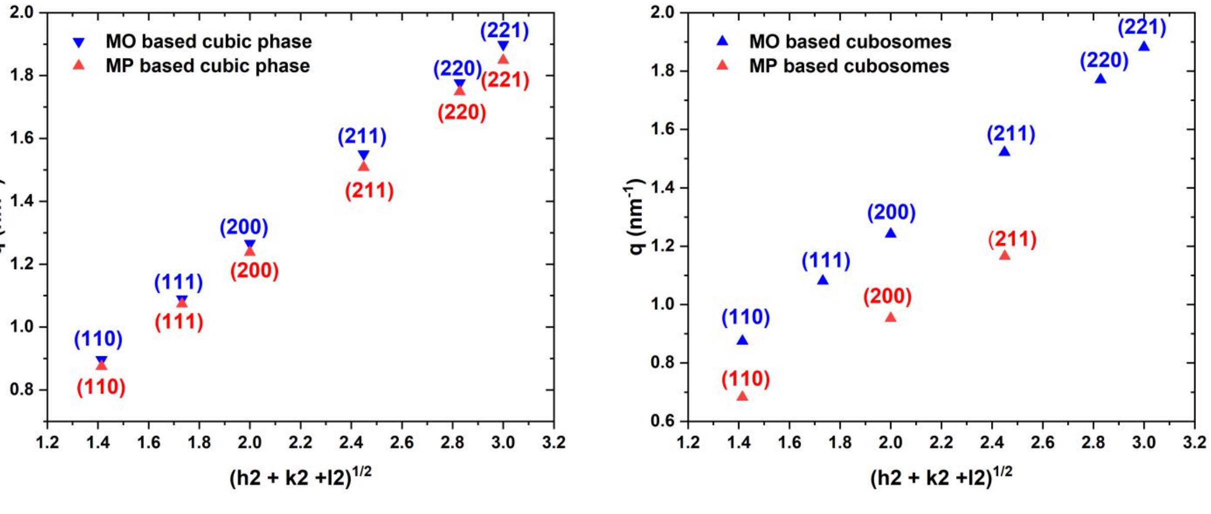

Figure S3. Plot of % cumulative drug release vs. time.

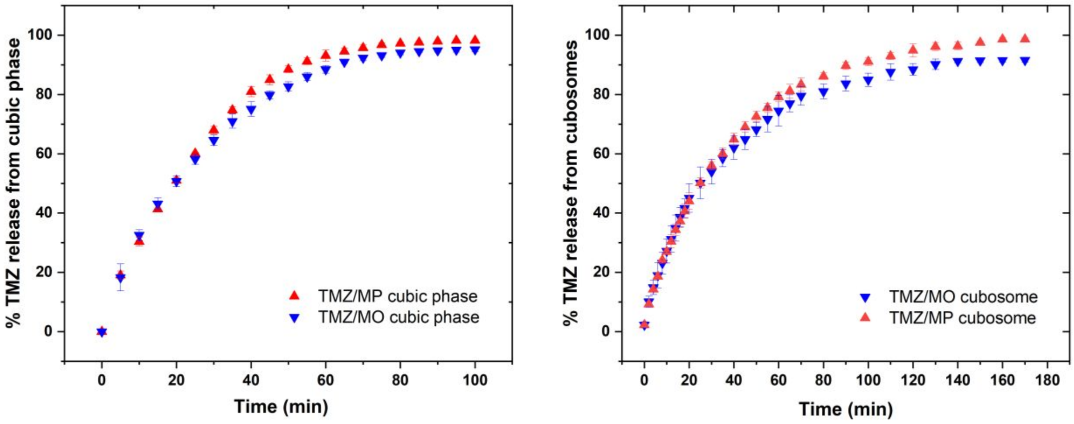

Figure S4. Analysis of the survival rate of A-172 (drug-sensitive) and T98G (drug-resistant) cells treated with non-loaded MO or MP phases determined using (A) MTS and (B) trypan blue exclusion assays. The number of viable, non-treated, cells was used as a control (100%). Data are presented as mean ± SD (standard deviation); (n = 8). For all analyzed comparisons $p > 0.05$.

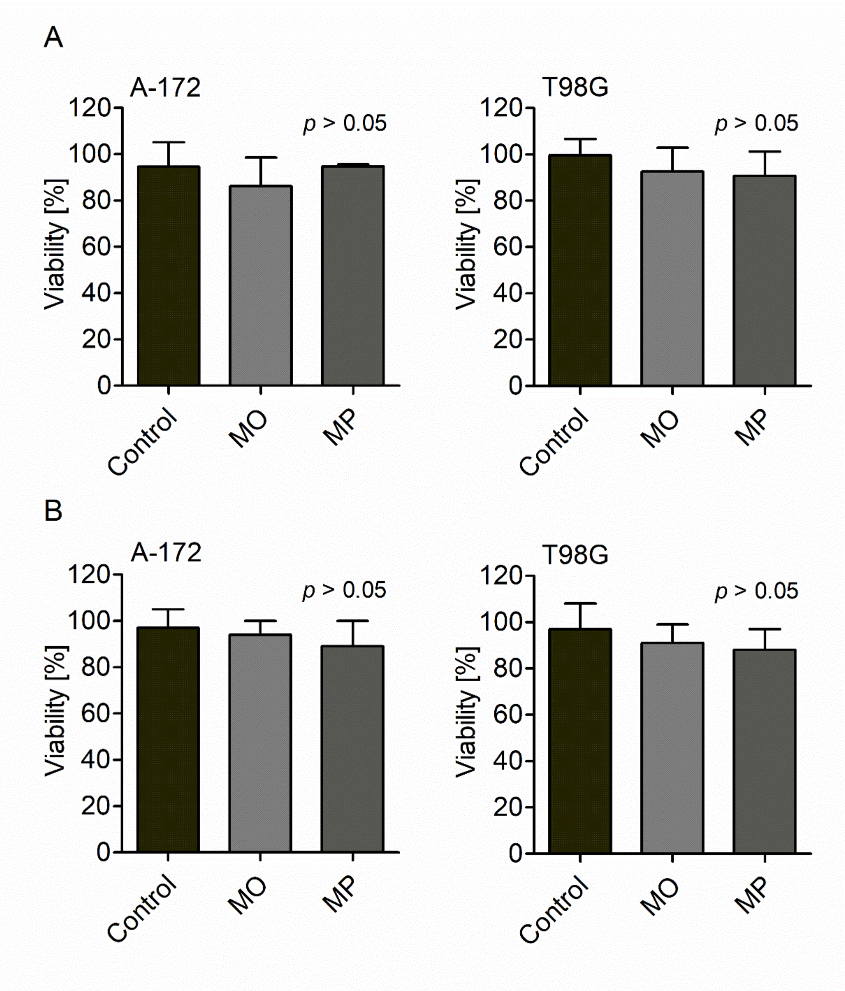

S2. Kinetic models.

The drug release data were fitted into Higuchi's equation: $M_t/M_\infty = k\sqrt{t}$, where M_t/M_∞ is the fraction of drug released at time *t*, and *k* is the release rate constant. In this model, the cumulative drug release is proportional to the square root of time. In the Korsmeyer-Peppas equation, $M_t/M_\infty = k_1\, t^n$, *k1* is a kinetic constant, *t* is the release time and *n* is the diffusional exponent. The diffusional exponent indicates the drug release mechanism. For n = 0.5 the drug release mechanism is Fickian diffusion, while for n = 1, Case II transport occurs. When the "n" value is between 0.5 and 1, it indicates anomalous transport.

The Peppas–Sahlin equation, which accounts for the coupled effects of Fickian diffusion and Case II transport, is: $M_t/M_\infty = k_1\, t^m + k_2\, t^{2m}$. The first term of this equation represents the contribution of Fickian diffusion while the second term refers to relaxation contribution.
